# Performance of the nonstructural 1 Antigen Rapid Test for detecting all four DENV serotypes in clinical specimens from Bangkok, Thailand

**DOI:** 10.1186/s12985-022-01904-0

**Published:** 2022-10-27

**Authors:** Kanaporn Poltep, Juthamas Phadungsombat, Nathamon Kosoltanapiwat, Borimas Hanboonkunupakarn, Witthawat Wiriyarat, Sarin Suwanpakdee, Phirom Prompiram, Emi E. Nakayama, Keita Suzuki, Hisahiko Iwamoto, Tatsuo Shioda, Pornsawan Leaungwutiwong

**Affiliations:** 1 Department of Microbiology and Immunology, Faculty of Tropical Medicine, Mahidol University, 420/6 Ratchawithi road, Ratchathewi, 10400 Bangkok, Thailand; 2 Mahidol-Osaka Center for Infectious Diseases (MOCID), Faculty of Tropical Medicine, Mahidol University, 420/6 Ratchawithi road, Ratchathewi, 10400 Bangkok, Thailand; 3 The Monitoring and Surveillance Center for Zoonotic Diseases in Wildlife and Exotic Animals (MoZWE), Faculty of Veterinary Science, Mahidol University, 999 Phutthamonthon Sai 4 Road, 73170 Phutthamonthon, Nakhonpathom, Thailand; 4 Center for Infectious Disease Education and Research (CiDER), Department of Viral Infections, Research Institute for Microbial Diseases (RIMD), Osaka University, 3-1, Yamada-oka, 565-0871 Suita, Osaka Japan; 5 Department of Clinical Tropical Medicine, Faculty of Tropical Medicine, Mahidol University, 420/6 Ratchawithi road, Ratchathewi, 10400 Bangkok, Thailand; 6 POCT Business Unit, TANAKA Kikinzoku Kogyo K.K, 2-73, 254-0076 Shinmachi, Hiratsuka, Kanagawa Japan

**Keywords:** Dengue, Diagnosis, NS1, ICT, RDT

## Abstract

**Background:**

Dengue is an arboviral disease that has a large effect on public health in subtropical and tropical countries. Rapid and accurate detection of dengue infection is necessary for diagnosis and disease management. We previously developed highly sensitive immunochromatographic devices, the TKK 1^st^ and TKK 2^nd^ kits, based on dengue virus (DENV) nonstructural protein 1 detection. However, these TKK kits were evaluated mainly using DENV type 2 clinical specimens collected in Bangladesh, and further validation using clinical specimens of other serotypes was needed.

**Methods:**

In the present study, one of the TKK kits, TKK 2^nd^, was evaluated using 10 DENV-1, 10 DENV-2, 4 DENV-3, 16 DENV-4, and 10 zika virus-infected clinical specimens collected in Bangkok, Thailand.

**Results:**

The TKK 2^nd^ kit successfully detected all four DENV serotypes in patient serum specimens and did not show any cross-reactivities against zika virus serum specimens. The IgM and/or IgG anti-DENV antibodies were detected in seven serum specimens, but did not seem to affect the results of antigen detection in the TKK 2^nd^ kit.

**Conclusion:**

The results showed that the TKK 2^nd^ kit successfully detected all four DENV serotypes in clinical specimens and confirmed the potential of the kit for dengue diagnosis in endemic countries.

**Supplementary Information:**

The online version contains supplementary material available at 10.1186/s12985-022-01904-0.

## Background

Dengue is a mosquito-borne viral infection. The symptoms vary from an acute undifferentiated febrile illness to the much more severe dengue shock syndrome which is found in a small number of patients with a heterotypic dengue virus (DENV) infection in endemic areas of tropical and subtropical regions [1, 2]. DENV infection impacts global human health due to the distribution of its main mosquito vector, *Aedes aegypti* [3], and human travel. DENV belongs to the genus *Flavivirus*, family *Flaviviridae*. DENV is an enveloped virus containing a single-stranded RNA, approximately 10.6 kb in length. DENV genomic RNA encodes three structural proteins (capsid (C), pre-membrane/membrane (prM/M), and envelope (E) proteins) and seven nonstructural (NS) proteins (NS1, NS2A, NS2B, NS3, NS4A, NS4B, and NS5) [4]. Based on antigenic differences, DENVs were divided into four serotypes, DENV-1, DENV-2, DENV-3, and DENV-4, each of which elicits only limited cross-protective immunity [4–6].

Nonstructural protein 1 (NS1) is a 48-kDa glycoprotein produced by all flaviviruses and is secreted from infected mammalian cells since the early phases of infection [7]. The NS1 protein was found in serum from the first day after the onset of clinical signs up to 9 days later. Furthermore, the viral NS1 protein can be detected even after defervescence, when viral nucleic acid is no longer detected [8]. NS1 levels during the acute phase correlate with the levels of viremia and disease severity [9–11]. Several studies have suggested that NS1 is a key mediator of the pathogenesis of flaviviruses and a representative biomarker for detection in the early phase of infection [12–14].

Immunochromatographic tests (ICTs) were developed for the detection of DENV NS1 antigen in approximately 10–15 min without specialized equipment or specialized personnel. An ideal rapid diagnostic test (RDT) has to reliably detect the infection, should be sensitive, specific, user-friendly, rapid and robust, equipment-free, and can be delivered to the field or hospitals for epidemiological studies with limited instruments [15]. However, the sensitivity and specificity or advantages and limitations of the test vary widely depending on the timing of specimen collection and various other factors in endemic areas [9, 16]. Recently, several commercial NS1 RDTs showed differing efficacies in the detection of different serotypes of DENV [17, 18]. We have previously reported the novel ICTs of DENV NS1 detection, the TKK 1^st^ and TKK 2^nd^ kits, and evaluated their efficiency in DENV clinical isolates and patient serum specimens in Bangladesh. The results showed higher efficiency and specificity of both of these kits for detection of DENV-1 genotype V, DENV-2 genotype Cosmopolitan, and DENV-3 genotype I than commercial ICT kits. However, we failed to evaluate these ICTs for DENV-4, which was not endemic in Bangladesh [19]. It would also be better to examine the kit in Thailand, where multiple DENV serotypes and genotypes co-circulate in the same area. In the present study, the aim was to evaluate one of the two TKK kits, the TKK 2^nd^ kit, in Thailand, since the TKK 2^nd^ kit showed slightly higher sensitivity than the TKK 1^st^ kit [19]. We report here that, the TKK 2^nd^ kit was found to show high levels of efficiency and specificity in clinical specimens of all four serotypes of DENV, which were collected and confirmed in the Hospital for Tropical Diseases, Bangkok, Thailand.

## Methods

The protocol of the present study was reviewed and approved by the Ethics Committee of the Faculty of Tropical Medicine, Mahidol University, Bangkok, Thailand under Ethical approval number TMEC 19–051. The present study was exempt from obtaining participants’ consent since only leftover specimens were used after anonymization. Serum specimens were obtained from anonymous DENV and zika virus (ZIKV)-positive patients who presented to the Hospital for Tropical Diseases during 2017–2020. The clinical serum specimens were separated and stored at − 80 °C until analysis.

Viral RNA was extracted from 70 µL of clinical serum specimens using a QIAamp viral RNA mini kit (Qiagen, Hilden, Germany) according to the manufacturer’s instructions. The viral RNA was eluted in 60 µL of nuclease-free water and stored at − 80 °C until analysis. The viral load data were measured by One-step SYBR Green I-based quantitative reverse transcriptase PCR (qRT-PCR) specific to DENV or ZIKV using previously described primer pairs and protocols [20, 21].For DENV-positive specimens, the viral load was expressed as plaque forming units per milliliter (PFU/mL) using serially diluted laboratory strains of DENV with known infectious titers as a standard curve. The extracted DENV RNA was also used for determination of DENV serotype using a commercial dengue subtyping multiplex kit (Genesig, Chandler’s Ford, UK) according to the manufacturer’s instructions.

A total of 30 µL of patient serum specimens was mixed with 60 µL of dilution buffer of the TKK 2^nd^ kit. The chromatographic stick was soaked in the mixture and incubated for 15 min at room temperature. The test band intensities were measured using a chromatogram reader (Hamamatsu photonics, model C10066-10). Bands with an intensity > 15 milli-absorbance units (mAbs) were visible by eye. The presence of anti-DENV IgM and IgG antibodies was detected using the SD-Bioline Duo kit (NS1Ag + IgG/IgM, The Alere Medical) according to the manufacturer’s instructions.

## Results

**Fig. 1:**
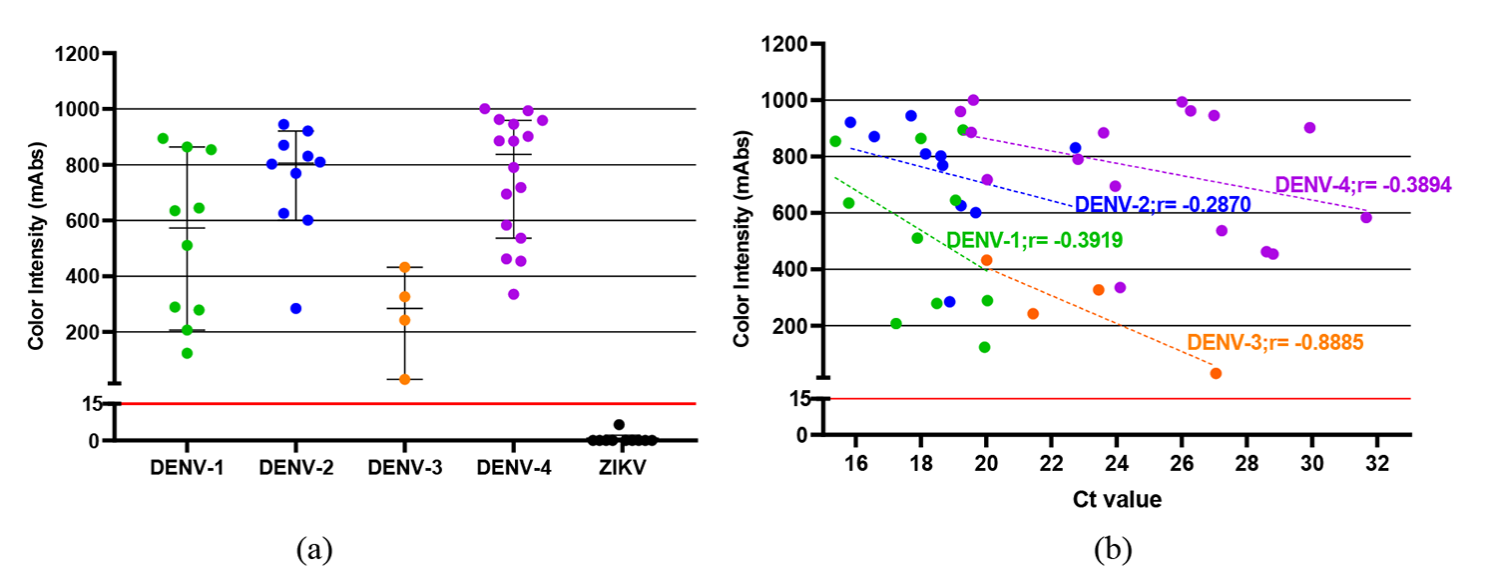
The DENV and ZIKV patient serum specimens from the Hospital for Tropical Diseases, Bangkok, Thailand, collected during 2017–2020, were used in the present study. The y-axis indicates color intensity quantified as milli-absorbance (mAbs) units using an immunochromatogram reader. Results obtained from the TKK 2^nd^ kit for all four DENV serotypes and ZIKV-positive specimens are shown. The green, blue, orange, purple, and black circles represent the DENV-1, 2, 3, 4, and ZIKV-positive serum specimens, respectively, with the median and interquartile range of color intensity (mAbs) (a). The dotted lines indicate linear regression of the correlation between Ct values and color intensity of the TKK 2^nd^ kit (b). The grid line at 15 mAbs on the y-axis is set as the cut-off value of the TKK 2^nd^ kit. Each of the data points represents the result of a single kit.

Forty RT-PCR confirmed DENV-positive serum specimens were obtained from the Hospital for Tropical Diseases during 2017–2020; there were 10 DENV-1, 10 DENV-2, 4 DENV-3, and 16 DENV-4 specimens. As negative controls, 10 ZIKV-positive serum specimens were used. The viral loads of DENV-positive specimens ranged from 1.34 × 10^3^ to 3.99 × 10^6^ PFU/mL, with a median of 3.70 × 10^5^ PFU/mL in all DENV-positive specimens. The median viral load of DENV-1-positive specimens was the highest among the four serotypes, followed by DENV-2, DENV-3, and DENV-4, in that order. The descriptive analysis comparing RDT results for DENV detection is shown in Table 1. The color intensity ranged from 30 to 1000.7 mAbs, with a median of 706.6 mAbs. The median color intensity of DENV-4 specimens was the highest among the four serotypes, followed by DENV-2, DENV-1, and DENV-3, in that order. The NS1 rapid antigen detection TKK 2^nd^ kit was positive in all these 40 DENV-positive serum specimens. The kit was negative in all 10 ZIKV-positive specimens. Therefore, the TKK 2nd kit exhibited 100% sensitivity, including serum specimens with a Ct value greater than 30 and five serum specimens with viral load less than 1 × 10^4^ PFU/mL. Including, there were seven serum specimens with IgM and/or IgG anti-DENV antibodies that were also positive with the TKK 2^nd^ kit. There were weak negative correlations between Ct value and color intensity (mAbs) in DENV-1, DENV-2, and DENV-4. In contrast, DENV-3 showed stronger negative correlation but none of them reached statistical significance (Table 1; Fig. 1). Specimen ID numbers, DENV serotype, genotype, and other data are shown in Additional file 1. It should be noted that the TKK 2^nd^ kit showed much higher color intensity than the SD Bioline RDT in 6 DENV-4 specimens (Additional file 1). Schematic illustrations and photographs of the TKK 2^nd^ kits used in the present study are shown in Additional file 2.

Moreover, specimens from 6 additional DENV-positive participants with a range of different time points during the infection were tested. Results showed that the TKK^2nd^ kit could detect DENV NS1 protein in serum specimens from clinical signs until defervescence in most of the participants (Additional file 3).

**Table 1:** Descriptive analysis of the TKK 2^nd^ kit for detection of DENV-positive clinical serum specimens

	DENV-1	DENV-2	DENV-3	DENV-4	All DENV-positive specimens
**Median viral load (Ct value)**	18.25	18.64	22.45	25.06	19.82
**Median viral load (PFU/mL)**	9.38 × 10^5^	8.65 × 10^5^	7.99 × 10^4^	2.57 × 10^4^	3.70 × 10^5^
**Median color intensity (mAbs)**	572.8	805.7	284.3	837.3	706.6
**no. of positive/no. of total specimens**	10/10	10/10	4/4	16/16	40/40
**% Sensitivity**	100	100	100	100	100
**Pearson r (Ct value– mAbs)**, **Two-tailed** ***P*** **value**	-0.3919, 0.2627	-0.2870, 0.4214	-0.8885, 0.1115	-0.3894, 0.136	-0.0945, 0.562

## Discussion

DENV is widespread in tropical and subtropical regions, and quick and simple detection methods would be beneficial for immediate responses in the early phase of dengue infection. High accuracy and efficiency for detection of all four DENV serotypes are important in dengue diagnosis. Although molecular detection of viral genomes using RT-PCR methods is the gold standard due to its highly sensitive and specific detection of the viruses, this technique requires expensive equipment and experienced personnel [13, 22]. So far, several NS1 antigen RDTs for DENV detection have been developed and released for early clinical diagnosis in hospitals and the field. High levels of sensitivity and specificity are essential for commercial RDTs [9, 16, 23]. In the present study, the sensitivity of the TKK 2^nd^ kit for DENV NS1 detection in PCR-confirmed positive sera was investigated in recent dengue patients in Bangkok, Thailand, where all four serotypes co-circulate. This TKK 2^nd^ kit demonstrated reasonable overall agreement with RT-PCR results for these specimens. In addition, the TKK 2^nd^ kit did not show any cross-reaction with serum specimens of ZIKA patients, confirming our previous results of laboratory strains of ZIKA [19]. Furthermore, the TKK 2^nd^ kit needs less serum volume than several commercial RDTs, which is another benefit for diagnosis in pediatric cases.

In addition, the TKK 2^nd^ kit was sensitive enough in both primary and secondary DENV infections, since the presence of anti-DENV antibodies did not seem to affect the result of antigen detection of the TKK 2^nd^ kit (Additional file 1). In contrast, several studies reported that the commercial NS1 RDTs produced false-negative results and low sensitivity in DENV secondary infections, and that antibodies against DENV NS1 in the patient specimens affected the results of their commercial RDTs, including a recently evaluated RDT in Thailand that showed 5 false-negative results [17, 24–28]. Thus, patients’ antibodies may eclipse the affinity of RDT antibodies by recognizing NS1 protein at the same epitope, leading to negative results [28–30]. However, ICT false-negative results may be caused by low levels of viremia and NS1 antigenemia during secondary infections [25, 31]. Therefore, the sensitivity and specificity of RDT in clinical specimens indicate the probability and capability of diagnostics [32].

Dengue is caused by four serotypes of DENV. Therefore, the commercial NS1 antigen test should detect all four DENV serotypes. The TKK 2^nd^ kit was able to detect these clinical specimens of all four serotypes of DENV, whereas previous RDT NS1 detection showed false-negative results due to amino acid variations of NS1 and secondary infections by DENV-2 strains [33–35]. Although DENV-2 was a predominant serotype in Malaysia in 2018, low sensitivity of DENV-2 by commercial RDT evaluation was recently reported [36]. On the other hand, the present study showed the ability of the TKK 2^nd^ kit to detect both Cosmopolitan and Asian I genotypes of DENV-2.

Moreover, the TKK 2^nd^ kit detected more efficiently for DENV-2 and DENV-4 than other serotypes (Fig. 1b). In contrast, the SD BIOLINE Dengue NS1 antigen kit was reported to show sensitivities less than 80% when compared with a newly developed serotyping NS1 ELISA system using DENV-2 and DENV-4 patient specimens in Thailand [37, 38]. Previous evaluations of both the commercial and newly developed RDT NS1 detection systems showed false-negative results and various levels of sensitivity [17, 24, 39–41]. A low level of NS1 protein in sera from DENV-3 and DENV-4 infections was reported, and the frequency of viremia of these serotypes was significantly lower than of other serotype infections [42–44]. Despite these findings, the present study showed that the TKK 2^nd^ kit was able to detect DENV infections with low levels of viremia in DENV-3 and DENV-4 cases (Fig. 1b).

Previous studies reported low sensitivity of a commercial ICT NS1 Ag test for detection of DENV-4 in serum specimens collected in Brazil, Thailand, Vietnam, and Malaysia [43, 45–49]. These studies suggest that lower sensitivity to DENV-4 than to the other three serotypes might be caused by monoclonal antibodies of commercial RDT which failed to capture epitopes of DENV-4 NS1 protein. Therefore, the present study showed further benefit of the TKK 2^nd^ kit for detecting DENV-4 that previously dominated in Thailand and has emerged in Laos. Anti-DENV antibody detection of either IgM or IgG would further increase the capability of dengue diagnosis [25, 50].

Our previous study reported performance of the TKK 1^st^ and 2^nd^ kits to detect NS1 proteins in clinical DENV isolates and patient serum specimens in Bangladesh and neither kit displayed any cross-reaction with other flaviviruses [19]. Consequently, the present study showed the performance of the TKK 2^nd^ kit in native NS1 proteins in clinical serum specimens in Bangkok, Thailand, where all four DENV serotypes currently co-circulate. Therefore, the present study confirmed the efficiency of the TKK 2^nd^ kit for detecting all four DENV serotypes distributed in Southeast Asia and Asia. Because DENV-3 was detected at very low prevalence during the sample collection period of the present study, we were able to evaluate only a small number of DENV-3 positive serum specimens. Nevertheless, this study exhibited that the TKK^2nd^ kit could detect both genotypes I and III of DENV-3 currently circulating in Bangkok, Thailand. Moreover, these results showed the improvement of the RDT for detection of all four DENV serotypes in clinical specimens. Such an ICT can be an effective RDT device for DENV detection that could be clinically useful in endemic countries. Although our results showed moderate levels of correlation between the levels of color intensities of the TKK^2nd^ kit and viral load, the color intensity merely indicated NS1 antigenemia and did not directly refer to the viremia levels in clinical serum specimens [19]. Nevertheless, numerous studies reported that DENV NS1-related disease status and serum NS1 levels showed significant differences between fatal and non-fatal cases [9–11, 13, 51]. Therefore, semi-quantitative NS1 level detection by the TKK 2^nd^ kit (Fig. 1b) may be used for monitoring or screening patients who might develop severe dengue symptom. However, further study is needed to evaluate this possibility.

There were DENV false-positive results in SARS-CoV-2 patients by antibody detection kits, which raised the possibility of cross-reactivity between DENV and SARS-CoV-2 [52, 53]. The effects of SARS-CoV-2 antigens and antibodies should also be carefully investigated using the TKK 2^nd^ kit in the future.

## Conclusion

The ICT TKK 2^nd^ kit, which has sufficient sensitivity and specificity for detection of DENV NS1 in the present study, would be valuable for rapid detection and management of all four serotypes of DENV in endemic areas.

## Electronic supplementary material

Below is the link to the electronic supplementary material.

Supplementary Material 1

Supplementary Material 2

Supplementary Material 3

## Data Availability

All data and materials used and/or analyzed during this study are included in this published article and its Additional files.

## Abbreviations

DENV: Dengue virus
ICT: Immunochromatographic test
NS1: Nonstructural protein 1
mAbs: Milli-absorbance
RDT: Rapid diagnostic test
