# Correction to: Performance of the nonstructural 1 Antigen Rapid Test for detecting all four DENV serotypes in clinical specimens from Bangkok, Thailand

**DOI:** 10.1186/s12985-023-02236-3

**Published:** 2023-11-10

**Authors:** Kanaporn Poltep, Juthamas Phadungsombat, Nathamon Kosoltanapiwat, Borimas Hanboonkunupakarn, Witthawat Wiriyarat, Sarin Suwanpakdee, Phirom Prompiram, Emi E. Nakayama, Keita Suzuki, Hisahiko Iwamoto, Tatsuo Shioda, Pornsawan Leaungwutiwong

**Affiliations:** 1 Department of Microbiology and Immunology, Faculty of Tropical Medicine, Mahidol University, 420/6 Ratchawithi road, 10400 Ratchathewi, Bangkok, Thailand; 2 Mahidol-Osaka Center for Infectious Diseases (MOCID), Faculty of Tropical Medicine, Mahidol University, 420/6 Ratchawithi road, 10400 Ratchathewi, Bangkok, Thailand; 3 The Monitoring and Surveillance Center for Zoonotic Diseases in Wildlife and Exotic Animals (MoZWE), Faculty of Veterinary Science, Mahidol University, 999 Phutthamonthon Sai 4 Road, 73170 Phutthamonthon, Nakhonpathom, Thailand; 4 Center for Infectious Disease Education and Research (CiDER), Department of Viral Infections, Research Institute for Microbial Diseases (RIMD), Osaka University, 3-1, Yamada-oka, 565-0871 Suita, Osaka Japan; 5 Department of Clinical Tropical Medicine, Faculty of Tropical Medicine, Mahidol University, 420/6 Ratchawithi road, 10400 Ratchathewi, Bangkok, Thailand; 6 POCT Business Unit, TANAKA Kikinzoku Kogyo K.K, 2-73, 254-0076 Shinmachi, Hiratsuka, Kanagawa Japan

**Correction to: Virology Journal (2022) 19:169**

10.1186/s12985-022-01904-0

Following publication of the original article [1], the authors informed us that would like to update the **Ethics approval and consent to participate** section.

The incorrect Ethics approval and consent to participate is:

The protocol of this study was reviewed and approved by the Ethics Committee of the Faculty of Tropical Medicine, Mahidol University, Bangkok, Thailand (TMEC 19–051). This study was exempt from patient consent since only leftover specimens were used after anonymization.

The correct Ethics approval and consent to participate is:

The protocol of this study was reviewed and approved by the Ethics Committee of the Faculty of Tropical Medicine, Mahidol University, Bangkok, Thailand (MUTM 2020-024-01). This study was exempt from patient consent since only leftover specimens were used after anonymization.
