# Supplementary material for: Performance of the nonstructural 1 Antigen Rapid Test for detecting all four DENV serotypes in clinical specimens from Bangkok, Thailand
Source: Virol J. 2022 Oct 27;19:169. doi: 10.1186/s12985-022-01904-0 (PMC9610331; doi:10.1186/s12985-022-01904-0)
Supplement: Supplementary file 1 — Supplementary Material 1 [file 12985_2022_1904_MOESM1_ESM.pdf]

Additional file 1: Clinical specimens used to evaluate the TKK 2nd kit.

Sample ID	Collection date	Ct value	PFU/ml	Serotype	Genotype	DENV IgM	DENV IgG	Color intensity of the TKK 2nd kit (mAbs)	Color intensity of SD Bioline RDT (mAbs)
DV1I-TM18-20	28-May-18	18.00	1.39E+06	1	I	NA	NA	863.9	NA
DV1I-TM19-70	23-Dec-19	15.38	3.99E+06	1	I	NA	NA	854.1	NA
DV1I-TM19-40	29-Nov-19	15.79	3.92E+06	1	I	NA	NA	635.2	NA
DV1I-TM19-52	10-Dec-19	19.06	5.19E+05	1	I	NA	NA	644.9	NA
DV1I-TM19-75	28-Dec-19	19.29	4.56E+05	1	I	NA	NA	894.0	NA
DV1I-TM19-24	22-Nov-19	20.04	2.73E+05	1	I	NA	NA	288.9	NA
DV1I-TM20-21	16-Feb-20	17.24	1.66E+06	1	I	Negative	Negative	207.2	NA
DV1I-TM20-47	19-Aug-20	17.89	1.11E+06	1	I	NA	NA	510.4	NA
DV1I-TM20-24	27-Jun-20	18.49	7.66E+05	1	I	NA	NA	278.8	NA
DV1I-TM20-25	5-Jul-20	19.95	3.10E+05	1	I	NA	NA	123.6	NA
DV2C-TM19-37	28-Nov-19	15.84	3.79E+06	2	Cosmopolitan	NA	NA	921.0	NA
DV2A-TM19-13	19-Nov-19	17.70	1.18E+06	2	Asian I	NA	NA	944.4	NA
DV2C-TM19-41	30-Nov-19	18.67	6.42E+05	2	Cosmopolitan	NA	NA	768.7	NA
DV2C-TM19-32	26-Nov-19	19.23	4.52E+05	2	Cosmopolitan	NA	NA	625.6	NA
DV2C-TM19-31	26-Nov-19	19.68	3.40E+05	2	Cosmopolitan	NA	NA	600.9	NA
DV2A-TM19-43	3-Dec-19	22.74	6.72E+04	2	Asian I	NA	NA	830.7	NA
DV2A-TM20-96	3-Nov-20	16.57	3.39E+06	2	Asian I	NA	NA	870.4	NA
DV2A-TM20-94	30-Oct-20	18.88	1.02E+06	2	Asian I	NA	NA	284.2	NA
DV2C-TM20-65	22-Sep-20	18.14	1.50E+06	2	Cosmopolitan	NA	NA	809.5	NA
DV2C-TM20-36	3-Aug-20	18.61	7.10E+05	2	Cosmopolitan	NA	NA	801.8	NA
DV3I-TM20-10	11-Jan-20	20.02	2.98E+05	3	I	NA	NA	431.9	NA
DV3I-TM20-02	2-Jan-20	21.44	1.24E+05	3	I	NA	NA	242.1	NA
DV3III-TM20-07	10-Jan-20	23.45	3.58E+04	3	III	NA	NA	326.5	NA
DV3I-TM20-09	11-Jan-20	27.05	3.86E+03	3	I	NA	NA	30.0	NA
DV4I-TM20-76	3-Oct-20	28.80	2.34E+03	4	I	NA	NA	453.7	NA
DV4I-TM18-7	27-Mar-18	22.82	1.20E+05	4	I	NA	NA	790.1	NA
DV4I-TM20-90	21-Oct-20	19.21	8.60E+05	4	I	Negative	Negative	959.2	NA
DV4I-TM20-41	14-Aug-20	19.54	4.00E+05	4	I	Negative	Positive	885.3	NA
DV4I-TM20-69	28-Sep-20	19.61	6.99E+05	4	I	NA	NA	1000.7	NA
DV4I-TM20-64	18-Sep-20	20.03	5.63E+05	4	I	Negative	Positive	718.3	NA
DV4I-TM20-59	11-Sep-20	24.11	6.76E+04	4	I	NA	NA	334.9	NA
DV4I-TM20-98	9-Nov-20	26.01	2.52E+04	4	I	Negative	Positive	993.3	NA
DV4I-TM20-53	29-Aug-20	26.27	2.20E+04	4	I	Positive	Positive	961.9	NA
DV4I-TM20-5	9-Jan-20	27.23	3.46E+03	4	I	NA	NA	536.5	NA
DV4I-TM20-27	11-Jul-20	23.60	3.25E+04	4	I	Negative	Positive	884.4	255.1
DV4I-TM20-28	13-Jul-20	23.96	2.61E+04	4	I	Negative	Negative	694.8	201.0
DV4I-TM20-34	2-Aug-20	28.60	1.49E+03	4	I	Negative	Negative	461.9	99.1
DV4I-TM20-76	3-Oct-20	31.66	1.34E+03	4	I	Positive	Positive	583.2	55.4
DV4I-TM18-77	11-Oct-18	26.99	1.45E+04	4	I	Negative	Negative	945.4	352.8
DV4I-TM18-48	14-Aug-18	29.93	3.27E+03	4	I	Negative	Positive	901.9	209.8
ZK-17	NA	28.21	NA	NA	NA	NA	NA	6.5	NA
ZK-19	NA	25.19	NA	NA	NA	NA	NA	0	NA
ZK-20	NA	28.09	NA	NA	NA	NA	NA	0	NA
ZK-24	NA	28.08	NA	NA	NA	NA	NA	0	NA
ZK-31	NA	34.23	NA	NA	NA	NA	NA	0	NA
ZK-32	NA	28.10	NA	NA	NA	NA	NA	0	NA
ZK-36	NA	28.32	NA	NA	NA	NA	NA	0	NA
ZK-38	NA	28.08	NA	NA	NA	NA	NA	0	NA
ZK-39	NA	26.28	NA	NA	NA	NA	NA	0	NA
ZK-47	NA	29.40	NA	NA	NA	NA	NA	0	NA

NA; Data not available
