# Supplementary material for: Performance of the nonstructural 1 Antigen Rapid Test for detecting all four DENV serotypes in clinical specimens from Bangkok, Thailand
Source: Virol J. 2022 Oct 27;19:169. doi: 10.1186/s12985-022-01904-0 (PMC9610331; doi:10.1186/s12985-022-01904-0)
Supplement: Supplementary file 2 — Supplementary Material 2 [file 12985_2022_1904_MOESM2_ESM.pdf]

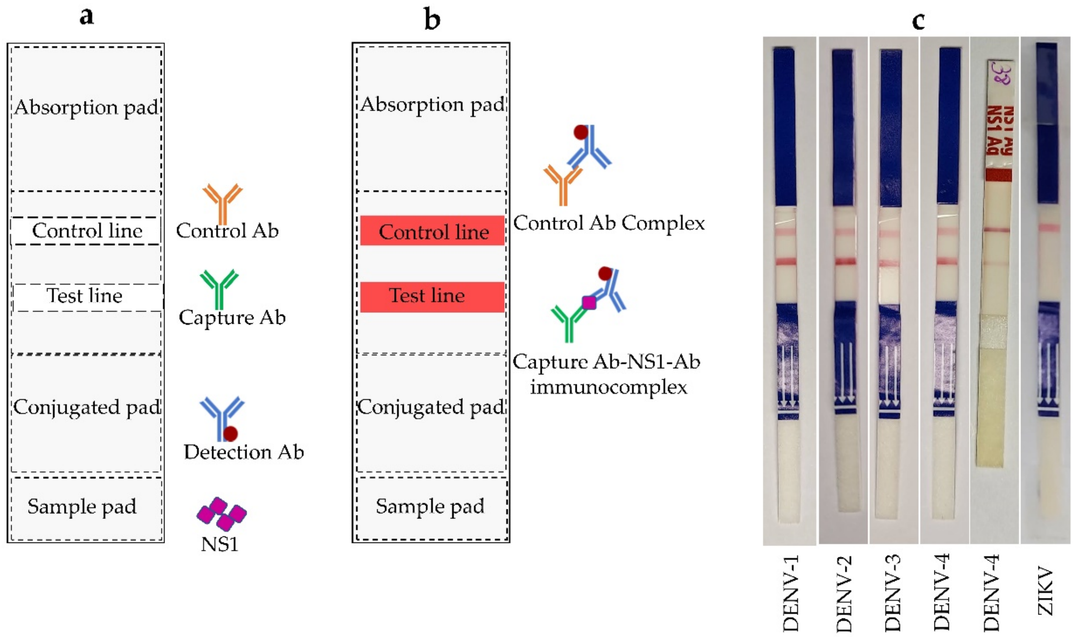

Additional file 2. Schematic illustrations and photographs of the TKK 2nd kit for detection of DENV NS1 proteins in DENV-positive clinical serum specimens. The TKK 2nd kit comprises the following elements: sample pad, the detection antibodies conjugated with colloidal gold nanoparticles on the conjugated pad, capture antibodies fixed on the test line of the membrane, control antibodies fixed on the control line, and the absorbance pad (a). The NS1 proteins are captured with conjugated antibodies. Subsequently, these complexes are bound with capture antibodies as the antibody-NS1-antibody complex at the test line. Unbound conjugated antibodies are captured with antibodies at the control line (b). Appearance of color at the test and control lines indicates positive results in clinical specimens with four DENV serotypes (DENV-1, DENV-2, DENV-3, and DENV-4). One DENV-4-positive result with the SD-Bioline NS1Ag kit (DENV-4) and a negative result with ZIKV-positive specimens in the TKK 2nd kit (ZIKAV) are also shown (c).
