# Supplementary material for: Performance of the nonstructural 1 Antigen Rapid Test for detecting all four DENV serotypes in clinical specimens from Bangkok, Thailand
Source: Virol J. 2022 Oct 27;19:169. doi: 10.1186/s12985-022-01904-0 (PMC9610331; doi:10.1186/s12985-022-01904-0)
Supplement: Supplementary file 3 — Supplementary Material 3 [file 12985_2022_1904_MOESM3_ESM.pdf]

Additional file 3: The performance of TKK^{2nd} kit in serum specimens of DENV-positive participants with a range of different time points during the infection

**Defervescence date**	**Project ID**	**Collection date**	**TKK 2nd kit**	
Day 6	BKK-002	Day 1	++[a]	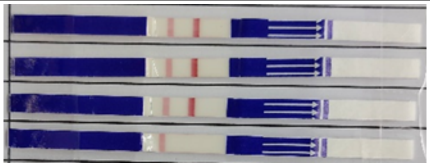
		Day 2	++	
		Day 4	++	
		Day 6	-[b]	
Day 5	BKK-003	Day 1	++	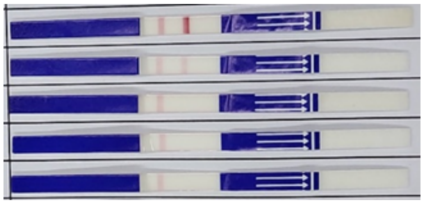
		Day 3	+[c]	
		Day 4	-	
		Day 5	-	
		Day 7	-	
Day 3	BKK-004	Day 1	++	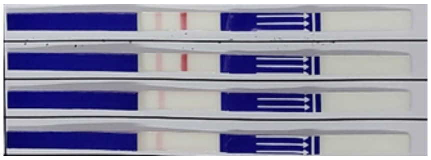
		Day 2	++	
		Day 5	-	
		Day 17	-	
Day 2	BKK-005	Day 1	++	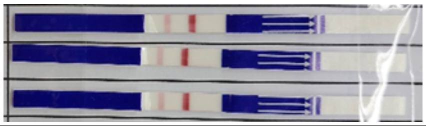
		Day 2	++	
		Day 3	++	
Day 2	BKK-006	Day 1	++	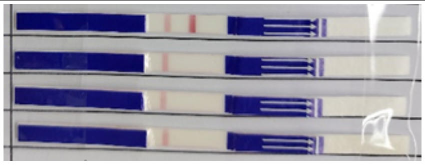
		Day 3	-	
		Day 4	-	
		Day 6	-	
Day 5	BKK-008	Day 1	++	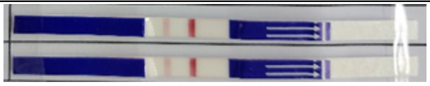
		Day 3	++	

a; The test band intensity was stronger than the control band intensity.
b; No visible test band appeared.
c; The test band intensity was equivalent to or weaker than the control band intensity.
